# Nicotine alleviates MPTP-induced nigrostriatal damage through modulation of JNK and ERK signaling pathways in the mice model of Parkinson’s disease

**DOI:** 10.3389/fphar.2023.1088957

**Published:** 2023-02-02

**Authors:** Sisi Ruan, Jiqing Xie, Linhai Wang, Lulu Guo, Yan Li, Wu Fan, Rongzhan Ji, Zhenlin Gong, Yan Xu, Jian Mao, Jianping Xie

**Affiliations:** ^1^ Flavour Science Research Center, College of Chemistry, Zhengzhou University, Zhengzhou, China; ^2^ Zhengzhou Tobacco Research Institute of CNTC, Zhengzhou, China; ^3^ Technology and Research Center, China Tobacco Jiangsu Industrial Co., Ltd.,, Nanjing, China; ^4^ Department of Medical Genetics and Cell Biology, School of Basic Medical Sciences, Zhengzhou University, Zhengzhou, China

**Keywords:** nicotine, neuroprotective effect, MAPK pathway, Parkinson’s disease, nigrostriatal region

## Abstract

**Introduction:** Nicotine (Nic) has previously been proven to reduce neurodegeneration in the models of Parkinson’s disease (PD). The present study is intended to investigate the detailed mechanisms related to the potential neuroprotective effects of Nic *in vivo*.

**Methods:** We established a PD model using 1-methyl-4-phenyl-1,2,3,6-tetrahydropyridine (MPTP)-induced C57BL6 mice (25 mg/kg/d, 5 d, i.p.) to investigate the neuropharmacological modulation of Nic pretreatment (2.5 mg/kg/d, 5 d, i.p., 30 min before MPTP injection) from the perspectives of neurobehavioral assessment, the pathological alterations, microglial cell inflammation and MAPK signaling pathways in specific brain regions.

**Results:** The open field test, elevated plus maze, rotarod and traction test suggested that Nic pretreatment could significantly improve MPTP-induced motor impairment and had an anxiolytic effect. Nic was found to improve neuroapoptosis, enhance tyrosine hydroxylase activity, and reduce the accumulation of the phosphorylated α-synuclein in the substantia nigra and striatal regions of PD mice by TUNEL and immunohistochemical assays. Immuno-fluorescent method for labeling Iba1 and CD68 indicated that Nic remarkably alleviates the activation of microglia which represents the M1 polarization state in the mice brain under MPTP stimulation. No significant difference in the expression of p38/MAPK pathway was found in the nigrostriatal regions, while Nic could significantly inhibit the elevated p-JNK/JNK ratio and increase the declined p-ERK/ERK ratio in the substantia nigra of MPTP-exposed brains, which was further confirmed by the pretreatment of CYP2A5 inhibitor to decline the metabolic activity of Nic.

**Discussion:** The molecular signaling mechanism by which Nic exerts its neuroprotective effects against PD may be achieved by regulating the JNK and ERK signaling pathways in the nigra-striatum related brain regions.

## 1 Introduction

Parkinson’s disease (PD) is one of the most common age-related multisystem neurodegenerative disorders, which is mainly characterized by the loss of dopaminergic neurons and the production of Lewy Bodies (LBs) and Lewy Neurites (LNs) made of misfolded alpha-synuclein (α-syn) (Wakabayashi, et al., 2013; Kalia, et al., 2015; Henderson, et al., 2019; Mehra, et al., 2019). Among these aggregates, phosphorylation of α-syn at the serine 129 site (p-α-syn) was the predominant pathological form (Colom-Cadena, et al., 2017; Hu, et al., 2020) until the patient was first diagnosed with a large deletion of dopaminergic neurons in the substantia nigra pars compacta (SNpc), where neurodegeneration had spread to other central nervous system (CNS) regions (DeWeerdt, 2016; Du, et al., 2021). Dopaminergic cell death decreases the release of dopamine (DA) in the striatum and other parts of the brain, which in turn leads to the motor deficits characterized by tremors, myotonic myopathies, and bradykinesia associated with this disorder (Bordia, et al., 2015; Acharya and Kim, 2021; Tizabi, et al., 2021). Non-motor symptoms can also be observed, such as autonomic disorders, anxiety, olfactory, cognitive and sleep disturbances (Schneider, et al., 2017; Balestrino and Schapira, 2020). Decades of research suggest that both genetic and environmental factors as well as interactions among them could trigger the widespread accumulation of toxic α-syn, oxidative stress, abnormal inflammation, mitochondria impairment and damage to nerve cells (Surmeier, et al., 2017; Mavroeidi and Xilouri, 2021). The pathological α-syn, neuroinflammation and microglia activation are considered to be hallmarks of PD (Yang, et al., 2018; Yang and Zhou, 2019; Ugalde-Muniz, et al., 2020). Therefore, discovering and developing medicines that have anti-inflammatory properties or antioxidant effects to reduce apoptosis and improve neuronal survival have been used as a strategy for the treatment of PD (Lev, et al., 2003; Lee, et al., 2019).

Epidemiological findings and numerous case-report suggest the decline in PD with tobacco use is a true biological effect (Ma, et al., 2020). In fact, nicotine (Nic) intake is negatively associated with the prevalence of PD, and Nic is thought to be a major mediator of neuroprotection according to extensive studies in experimental animal models (Barreto, et al., 2015). Nic reduces oxidative stress and neuroinflammation in the mouse brain and increases synaptic plasticity and survival of dopaminergic neurons, effects that improve mood, motor skills and memory (Nicholatos, et al., 2018; Alhowail, 2021). It has been shown that Nic may exert neuroprotective effects through α7 nicotinic acetylcholine receptor (α7-nAChRs) mediated anti-inflammatory effects (Park, et al., 2007) and α-syn clearance (Zhao, et al., 2021). Although a large body of experimental data supports the neuroprotective effects of Nic in the models of PD (Quik, et al., 2012; Quik, et al., 2015; Cai, et al., 2017; Nicholatos, et al., 2018), the exact signaling mechanism remains unclear.

Mitogen-activated protein kinases (MAPKs), which mainly include c-Jun NH_2_-terminal kinase (JNK), p38 MAPK, and extracellular signal-regulated kinase (ERK) in mammals (Harper and Wilkie, 2003), mediate intracellular signals associated with a variety of cellular activities, such as proliferation, differentiation, apoptosis, survival, inflammation, and innate immunity (Kim and Choi, 2015; Gui, et al., 2020). Previous studies have shown that the impaired MAPK signaling pathways plays a key role in the pathogenesis of various diseases, including cancer and neurodegenerative diseases (Kim and Choi, 2010; Rai, et al., 2019). For instance, the JNK signaling pathway was proved to be associated with the progressive loss of dopaminergic neurons in the substantia nigra (Zhao, et al., 2022). *In vitro* study using SH-SY5Y cells challenged with 1-methyl-4-phenylpyridinium (MPP^+^) showed that Nic pretreatment protected apoptotic cell death through activation of α7-nAChRs/MAPK/p53 axis, and the ERK signaling pathway might play an important role in the neuroprotective effects of α7-nAChRs (Xu, et al., 2019). However, whether Nic can exert neuroprotective effects through modulation of MAPK signaling pathways *in vivo* has not been very clear, and it is intriguing to determine which MAPK signaling pathway partakes in the anti-apoptotic process with Nic pretreatment.

In the present study, we investigated the neuroprotective effects of Nic on PD using a 1-methyl-4-phenyl-1,2,3,6-tetrahydropyridine (MPTP)-treated C57BL/6 mouse model (Zhang, et al., 2017). It was found that Nic pretreatment ameliorated MPTP-induced locomotor deficit and anxiety-like behavior in PD mice. Moreover, Nic pretreatment could also alleviate cell apoptosis, protect dopaminergic neuronal damage, reduce p-α-syn accumulation, and inhibit microglia activation as well as the expression of pro-inflammatory factors in substantia nigra and striatum of the PD mice model. The molecular signaling mechanism by which Nic exerts this neuroprotective effect may be achieved by regulating the JNK and ERK, but not p38, signaling pathways in the nigrostriatal regions, which was further confirmed by the pretreatment of CYP2A5 inhibitor to delay the metabolic process of Nic.

## 2 Materials and methods

### 2.1 Reagents and antibodies

MPTP hydrochloride (Bidepharm, BD59516, China), Nicotine (Toronto Research Chemicals, N412420, Canada), Nictione-d_4_ (Toronto Research Chemicals, N412427, Canada) and Cotinine (Cot) (Toronto Research Chemicals, C725000, Canada), 5-Methoxypsoralen (5-MOP) (Sigma, 275727, United States), Corn oil (Macklin, C805618, China), Chloral hydrate (Aladdin, C104202, China), Mounting Medium, antifading (with DAPI) (Solarbio, S2110, China), Protease Inhibitor Cocktail (MCE, HY-K0010, United States), Phosphatase Inhibitor Cocktail II (MCE, HY-K0022, United States), PMSF (Solarbio, P0100, China), RIPA buffer (high) (Solarbio, R0010, China).

All antibodies used in this study were as follows: pi-α-synuclein (Wako, 015-25191, Japan), Anti-Tyrosine Hydroxylase (Millipore, AB152, United States), Anti-iba1 (Wako, 019-19741, Japan), Anti-CD68 (BioLegend, 137001, United States), Goat anti-Mouse IgG-FITC (Absin, 20003, China), Goat anti-Rabbit IgG-FITC (ZSGB-BIO, ZF-0311, China), Goat anti-Mouse IgG-TRITC (ZSGB-BIO, ZF-0313, China), Goat anti-Rabbit IgG-TRITC (ZSGB-BIO, ZF-0316, China), iNOS (D6B6S) Rabbit mAb (Cell Signaling Technology, 13120, United States), Anti-IL-6 (abcam, ab229381, Uinted Kingdom), SAPK/JNK antibody (Cell Signaling Technology, 9252, United States), Phospho-SAPK/JNK (Thr183/Tyr185) (Cell Signaling Technology, 4668, United States), p38 MAPK (D13E1) (Cell Signaling Technology, 8690, United States), Phospho-p38 MAPK (Thr180/Tyr182) (Cell Signaling Technology, 4511, United States), p44/42 MAPK (Erk1/2) (Cell Signaling Technology, 4695, United States), Phospho-p44/42 MAPK (Erk1/2) (Cell Signaling Technology, 4370, United States). All secondary antibodies used for immunoblot analysis were obtained from Protein Simple (United States).

### 2.2 Animal experiments

Male C57BL/6 mice, 6–8 weeks old, with a body mass of 20 ± 2 g, were used for the experiments and were provided by the Henan Huaxing Experimental Animal Center (License number SCXY(Yu) 20190002, Zhengzhou, China). All mice were housed in an environment with a 12 h light/dark cycle at 22°C ± 1°C with available food and water. Animal experimental protocols conformed to the Guidelines for the Care and Use of Laboratory Animals and was granted by the Life Science Ethics Review Committee of Zhengzhou University.

**FIGURE 1:**
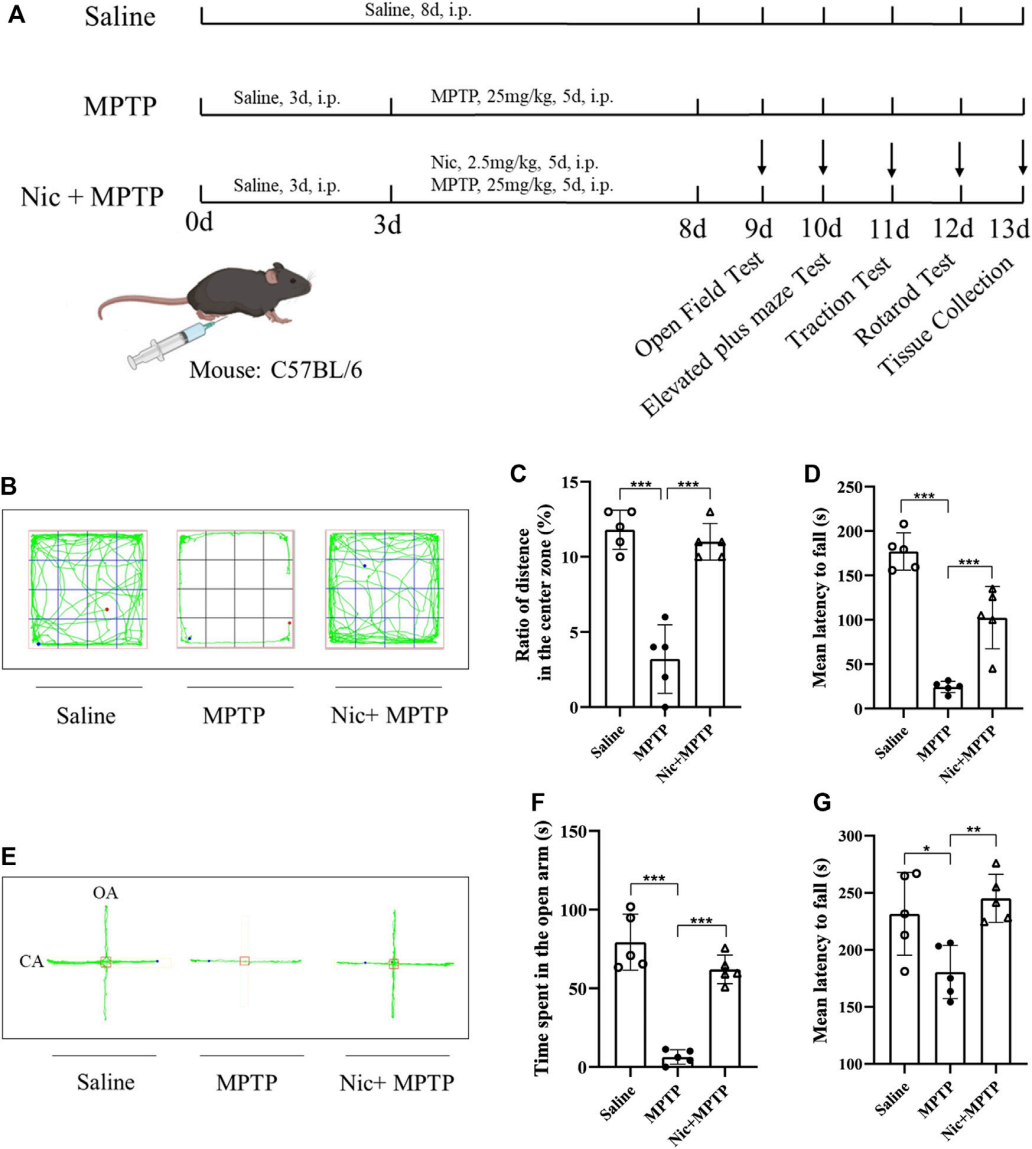
Effects of nicotine on locomotor performance and anxiety-like behavior in MPTP-treated mice. **(A)** Schematic diagram of the construction of a mouse model based on MPTP-induced neurological damage. **(B)** Representative locomotor paths of each group of mice in the open field test. **(C)** Percentage of horizontal motion distance in the central region in the open field test. **(D)** Mean latency to fall time in traction test. **(E)** Representative locomotor paths of each group of mice in the elevated plus maze test. **(F)** Open arm horizontal movement time in elevated plus maze. **(G)** Mean latency to fall time in the rotarod test. Data are expressed as mean ± SEM from five mice in each group. Significant differences were determined by an unpaired Student’s t-test and one-way ANOVA followed by Tukey’s multiple comparison *post hoc* test. *p < 0.05, **p < 0.01, ***p < 0.001.

Mice in the MPTP group (*n* = 10) were given saline for 3 days before receiving intraperitoneal injection of MPTP (25 mg/kg once daily for 5 days), while those in the control group (*n* = 10) were given saline (i.p.,) only for a total of 8 days. For the Nic and MPTP-treated group (Nic + MPTP) mice (*n* = 10) were given saline for 3 days before receiving Nic (2.5 mg/kg once daily, i.p.) (Quik, et al., 2009) and MPTP (30 min after Nic injection, i.p.) treatment for a total of 5 days (Figure 1A). In order to regulate the metabolic process of Nic and further investigate the effects of Nic on MPTP-treated mice, animals in the MOP + Nic + MPTP group (*n* = 9) received intragastric administration of 5-MOP (20 mg/kg once daily) for 3 days (Damaj, et al., 2007) and then treated with Nic and MPTP (30 min after Nic injection, i.p.) for a total of 5 days.

### 2.3 Behavioral tests

#### 2.3.1 Open field test

The mice were first allowed to adapt to the environment of the experimental room for 30 min in advance, and then placed in the center of the test chamber (45 cm × 45 cm × 45 cm) with the video recorder above, and allowed to move freely for 1 min. During the test, the trajectory of mice exploring under dim light for 5 min was recorded using SuperMaze software (Xinruan Information Technology Co., Ltd., China). Before and after each test, the instrument needs to be wiped with 75% ethanol to eliminate animal odor.

#### 2.3.2 Elevated plus maze test

The elevated plus maze test was further performed to assess the anxiety-like behavior of mice. Before the formal test, the animals were allowed to adapt to the environment of the experimental room for 30 min in advance, and then the mice were placed in an elevated cross-maze mold with an open arm size of 5 cm × 50 cm, closed arm size of 5 cm × 50 cm × 10 cm and a central junction area of 5 cm × 5 cm equipped with a video camera above, and allowed to move freely for 1 min. The trajectory of mice exploring under dim light for 5 min during the test was recorded using SuperMaze software. The animal odor was eliminated with 75% ethanol before each test.

#### 2.3.3 Traction test

A traction test was performed to assess the muscular strength and motor coordination of the mice. The two front paws of the mice were placed smoothly on a smooth wire of 50 cm in height, 1.5 mm in diameter and 30 cm in length, and after confirming that the mice grasped it tightly, the falling time from the time of releasing to the mice within 5 min was recorded, and the test was repeated three times for each mouse, and the average value was taken.

#### 2.3.4 Rotarod test

We applied the rotarod test to further assess the locomotor abilities such as motor coordination, balance, and motor learning in mice using an automated rotarod (RWD, China). The day before the test, mice were trained at a constant speed of 10 rpm/min for 20 min. During the second day of testing, the mice were placed on an accelerated rotating bar device and the speed was increased from 5 rpm/min to 40 rpm/min uniformly over a period of 3 min and then held at 40 rpm/min to 5 min. The instrument will automatically record the drop time during the test, and the motion test data is taken as the average of the three test drop times.

### 2.4 Immunostaining

Animals were anesthetized with 5% Chloral hydrate (10 μL/g), and perfused with saline, then brains were separated and fixed with 4% paraformaldehyde, dehydrated with 15% and 30% sucrose, and embedded in optimal cutting temperature compound (OCT). OCT coronal sections were made 25 μm thick using a frozen sectioning machine (Leica, Germany) and stored in −20°C cryoprotective solution for further analysis.

The distribution of TH-positive neurons in the substantia nigra and striatum was observed by immunohistochemistry. Frozen sections were rinsed with PBST for 15 min and then treated with 3% H_2_O_2_ for 10 min. Next, sections were treated with PBST for 15 min and blocked with 5% BSA for 1 h at room temperature. Afterward, the sections were incubated with rabbit anti-TH antibody at 4°C for 12 h and rinsed with PBST for 15 min. Then stained using the SABC (Rabbit IgG)-POD Kit (Solarbio, SA0021, China). Finally, the slices were hematoxylin counterstained, dehydrated with an ethanol gradient, permeabilized with dimethyl benzene, and sealed with neutral balsam. These slices are dried and then photographed using Motic EasyScan (Motic, China).

Immunofluorescence techniques were applied to observe the expression of TH, p-α-syn, iba1, and CD68 in the substantia nigra and striatum. Frozen sections were rinsed with PBST for 30 min and then blocked with 5% BSA for 2 h at room temperature. Next, incubated with mouse anti-p-α-syn antibody, rabbit anti-TH antibody, rabbit anti-iba1 antibody, and mouse anti-CD68 antibody at 4°C for 14 h. After that, it was rinsed with PBST for 15 min and incubated with sheep anti-mouse IgG (FITC), sheep anti-rabbit IgG (FITC), sheep anti-mouse IgG (TRITC), and sheep anti-rabbit IgG (TRITC) for 2 h at room temperature and protected from light. Finally, the sections were rinsed with PBST for 15 min and transferred to slides with a soft brush, blotted dry, dripped with anti-fluorescence quencher containing DAPI, covered with coverslips, and sealed with nail polish. These photographs were taken using the Leica TCS SP8 single two-photon laser confocal system (Leica, Germany) to capture the field of view of the target area.

### 2.5 Tunel assay

Terminal deoxynucleotidyl transferase dUTP nick-end labeling (TUNEL) staining was applied to observe the distribution of apoptotic cells in the substantia nigra and striatum. Frozen sections were rinsed with PBST and sections were transferred to slides with a soft bristle brush. Staining was then performed using the kit TUNEL Apoptosis Detection Kit (Abbkine, KTA 2010, United States). Afterward, incubate for 1 h in a wet box at 37°C in a thermostat, protected from light, and rinse with PBST. Finally, the films were rinsed with ddH_2_O and blocked with Mounting Medium, antifading (with DAPI). These photographs were taken using the Leica TCS SP8 single two-photon laser confocal system to capture the field of view of the target area.

### 2.6 Immunoblot analysis

The substantia nigra and striatal tissues were quickly removed from the mice brain after anesthesia execution and homogenized in lysis buffer Protease Inhibitor Cocktail, Phosphatase Inhibitor Cocktail II, PMSF, and RIPA buffer (high). Protein concentrations were determined by using the BCA Protein Assay Kit (Solarbio, PC0020, China). The sample protein concentration was diluted to 2 μg/μL using a sample preparation kit (Protein Simple, United States), and capillary electrophoresis separation and protein detection were performed using the JESS system (Protein Simple, United States). The sample stock solution was mixed with 0.1× sample buffer and 5× master Mix, and the prepared samples were denatured on a metal bath at 95°C for 5 min, and then cooled on ice for 5 min after denaturation. The biotinylated ladder, prepared samples, antibody diluent II, primary antibodies, secondary antibodies, streptavidin-HRP, luminol-peroxide mix were assigned to the corresponding wells of the analysis plate. The corresponding wells of the analysis plate were placed in the JESS device for analysis. The results were analyzed quantitatively and statistically using Compass software (Protein Simple, United States).

### 2.7 Preparation of plasma samples and UHPLC-MS/MS analysis

Saline and MOP-pretreated mice were randomly grouped into five groups, respectively, with four mice in each group. At 10, 30, 60, 120, and 180 min after the administration of Nic (2.5 mg/kg, i.p.), blood was collected by removing eyeballs, and plasma was prepared as follows: 500 μL blood sample was centrifuged at 2000 rpm for 10 min, 100 μL supernatant was transferred into a new centrifuge tube, and then 20 μL 2 μg/mL nicotine-d_4_ and 280 μL acetonitrile was added. After vortex mixing, all samples were centrifuged again at 2000 rpm for 10 min, and the final supernatant was filtered with 0.22 μm microporous membrane for UHPLC-MS/MS analysis. Standard working solution was prepared by diluted stocking solution of Nic, cotinine and nicotine-d_4_ (10 mg/mL) with acetonitrile step by step. The calibration curve was obtained by analyzing standard mix working solution: 1, 5, 10, 25, 50, 100, 250 ng/mL, each concentration was repeatedly analyzed three times.

Nic, Cot and nicotine-d_4_ were analyzed on a UHPLC equipped with a high-resolution Q Extractive mass spectrometer (HRMS, Thermo Fisher Scientific, Germany). The separation was conducted on the ACQUITY UPLC BEH HILIC column (2.1 × 150 mm, 1.7 μm) (Waters Associates) with binary gradient elution. The mobile phase A was 10 mM ammonium formate aqueous solution with formic acid (0.1%, v/v), and B was acetonitrile. The flow rate was set at 0.2 mL/min. The injection volume was 5 μL, and the gradient elution was as follows: 0–2 min, 90%–80% B; 2–5 min, 80%–65% B, 5–7 min; 65% B; 7–8 min; 65%–95% B and 8–10 min, 95% B. The mass spectrometer was conducted in the parallel reaction monitoring mode to acquire two transitions for Nic, Cot and nicotine-d_4_, and the parameters were as follows: spray voltage at 3.5 kV, ion transfer tube temperature at 300°C; the sheath gas, auxiliary gas and S-lens RF level were set to 30, 3 (arb) and 60 V, respectively; the full MS scan range was 80–300 m/z with a resolution of 70000, and the automatic gain control (AGC) target was set at 1.0 e^5^ with a maximum injection time (IT) of 100 ms. For the MS^2^ scan, the mass resolution was set at 17500, AGC target at 1.0 e^5^, maximum IT 100 ms, isolation window 1.0 m/z. The normalized collision energy (NCE) for Nic, Cot and nicotine-d_4_ was set at 55%, 60%, and 55%, respectively.

### 2.8 Statistical analysis

The results of immunohistochemistry and immunofluorescence were measured using Image J (Fiji) software, and the resulting data were statistically analyzed and plotted in GraphPad Prism 8.0 software. All data were expressed as mean ± standard error of the mean (Mean ± SEM). Differences between means were analyzed by one-way ANOVA using GraphPad Prism 8.0, followed by Tukey’s multiple comparison *post hoc* test. Where appropriate, differences between the two groups were analyzed using unpaired Student's t-tests. In all values, *p* < 0.05 was considered statistically significant.

## 3 Results

### 3.1 Nicotine pretreatment ameliorates anxiety-like behavior and locomotor deficit in MPTP-induced mice

To assess anxiety-like behavior and locomotor performance after Nic treatment, several behavioral tests were conducted on mice according to the experimental design paradigm shown in Figure 1A. In the open field test, the ratio of the distance moved in the central zone to the total distance was used to assess the spontaneous activity and exploratory behavior of the mice. We found that the ratio was significantly lower in MPTP-treated mice compared to the saline group (*p* < 0.001). And this deficiency was prominently improved in mice in the Nic + MPTP group (*p* < 0.001) (Figures 1B, C). In addition, we also applied the elevated plus maze test (Figures 1E, F) to further investigate the anxiety-like behavior of mice, and the time of mice entering the open arm was thought to negatively correlate with the anxiety state. The results showed that MPTP-treated mice took significantly less time to enter the open arm compared to the saline group (*p* < 0.001), and the abnormality was significantly ameliorated by Nic pretreatment (*p* < 0.001) (Figure 1F).

Moreover, in order to observe the basic motor abilities such as muscle strength on balance, motor coordination and locomotor ability after Nic and MPTP treatment, we examined the traction and rotarod tests on mice. The results of the traction test (Figure 1D) showed extremely significantly lessened mean latency to fall time in MPTP-treated mice compared to the saline group (*p* < 0.001), and the time was significantly increased after Nic pretreatment (*p* < 0.001). Besides, as compared to the saline group, the rotarod tests revealed that the mean latency to fall time decreased dramatically in the MPTP group (*p* < 0.05). Whereas the Nic pretreatment prolonged the in-stick time of mice significantly (*p* < 0.01) which is comparable to normal levels (Figure 1G).

These results suggest that Nic pretreatment significantly ameliorates the anxiety-like behavior and locomotor deficit in mice caused by MPTP exposure.

### 3.2 Nicotine pretreatment alleviates MPTP-induced apoptosis in substantia nigra and striatum

**FIGURE 2:**
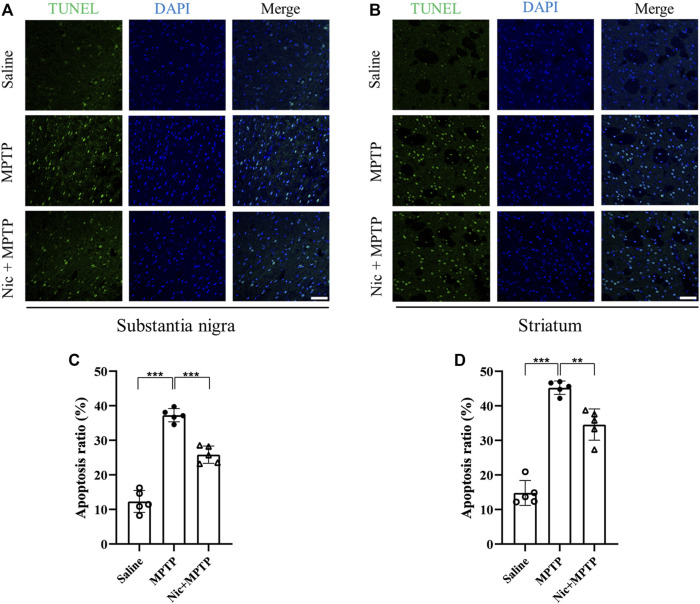
Effects of nicotine on apoptosis in the substantia nigra and striatal brain regions of MPTP-treated mice. Representative image of TUNEL signals (green panel), cell nuclei (blue panel) and co-localization (merge panel) in brain sections from the substantia nigra **(A)** and striatal regions **(B)** of mice. Statistical results on the status of apoptotic cells in TUNEL in the substantia nigra **(C)** and the striatal regions **(D)**. Scale bar at 50 μm. Data are expressed as mean ± SEM from five mice in each group, every point on the graph is an average from four fixed fields of view for each mouse. Significant differences were determined by an unpaired Student’s t-test and one-way ANOVA followed by Tukey’s multiple comparison *post hoc* test *p < 0.05, **p < 0.01, ***p < 0.001.

To specifically evaluate the apoptotic cells in the corresponding brain regions relating to PD *in vivo*, TUNEL-positive cells were detected and the co-localization analysis with nuclei indicated by DAPI was also performed. The results showed that in the substantia nigra (Figures 2A, C) and striatum (Figures 2B, D) of the mice brain, MPTP exposure induced severe apoptosis compared with the saline group (*p* < 0.001,*p* < 0.001), and the Nic pretreatment significantly mitigated this effect (*p* < 0.001, *p* < 0.01). These results indicate that Nic pretreatment can alleviate MPTP-induced cell apoptosis in the substantia nigra and striatal regions of the PD brain respectively.

### 3.3 Nicotine pretreatment protected against MPTP-induced damage to dopaminergic neurons and reduced the accumulation of the p-α-syn

**FIGURE 3:**
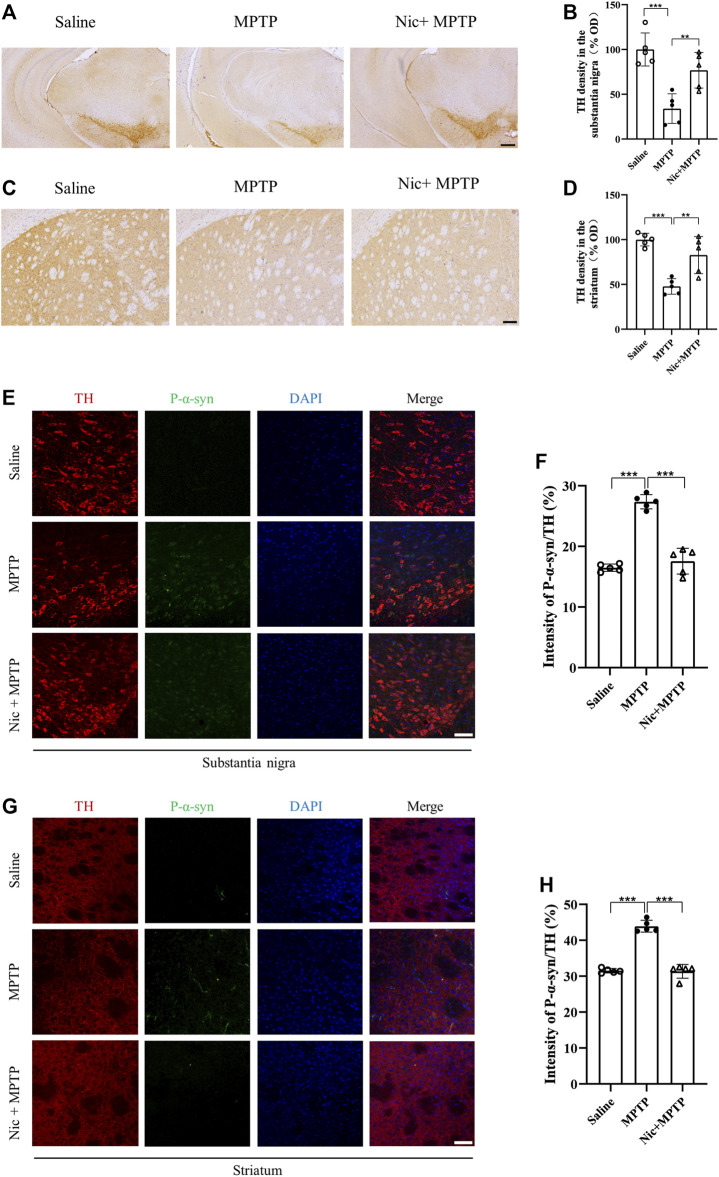
Effects of nicotine on dopaminergic neurons and p-α-syn expression in the substantia nigra and striatal brain regions of MPTP-treated mice. **(A)** Immunohistochemical staining results of TH-positive neurons in the midbrain. Scale bar at 300 μm. **(B)** Statistical map of TH-positive neurons density. **(C)** Immunohistochemical staining results of TH expression in striatal regions. Scale bar at 100 μm. **(D)** Statistical map of TH expression density. **(E)** Immunofluorescence histochemical staining results of tissue sections in the substantia nigra. Scale bar at 100 μm. **(F)** Statistical results of p-α-syn fluorescence intensity to TH ratio in the substantia nigra. **(G)** Immunofluorescence histochemical staining results of the striatal tissue sections. Scale bar at 100 μm. **(H)** Statistical results of p-α-syn fluorescence intensity to TH ratio in the striatal regions. Data are expressed as mean ± SEM from five mice in each group, every point on the graph is an average from four fixed fields of view for each mouse. Significant differences were determined by an unpaired Student’s t-test and one-way ANOVA followed by Tukey’s multiple comparison *post hoc* test. *p < 0.05, **p < 0.01, ***p < 0.001.

To determine whether Nic could exactly protect against dopaminergic neuronal loss and the pathological p-α-syn accumulation which are pathological hallmarks in PD, immunostaining of TH in the substantia nigra and the striatum was performed. The results showed (Figures 3A, C) that compared with the saline group, the MPTP group mice had a large number of TH-positive dopaminergic neuron deletions in the substantia nigra and lighter TH protein staining in the striatal regions, which were alleviated by Nic treatment. Further statistical results showed (Figures 3B, D) that optical density (OD) values were significantly lower in MPTP-treated mice (*p* < 0.001, *p* < 0.01), which was attenuated by Nic treatment (*p* < 0.001, *p* < 0.01).

The misfolding and aggregation of α-syn is an important marker for the development of PD, and p-α-syn accounts for one of the major parts of pathological post-translational modifications. We therefore used immunofluorescence staining to detect p-α-syn in response to pathological changes in the PD mice model, where p-α-syn expression was measured by the ratio of p-α-syn to TH. Immunostaining results showed (Figures 3E, G) that mice in the MPTP group had an elevated expression of p-α-syn in the substantia nigra and striatal regions. The ratio of p-α-syn to TH was significantly higher in MPTP-treated mice (*p* < 0.001), which was remarkably attenuated by Nic treatment (*p* < 0.001) (Figures 3F, H). All the results suggest that Nic pretreatment can further protect against MPTP-induced dopaminergic neuronal damage and reduce the accumulation of the pathological protein p-α-syn in the nigrostriatal pathway.

### 3.4 Nicotine pretreatment inhibits MPTP-induced activation of microglia and the expression of the pro-inflammatory factors iNOS and IL-6, which represent the M1 polarization state in the nigrostriatal pathway

**FIGURE 4:**
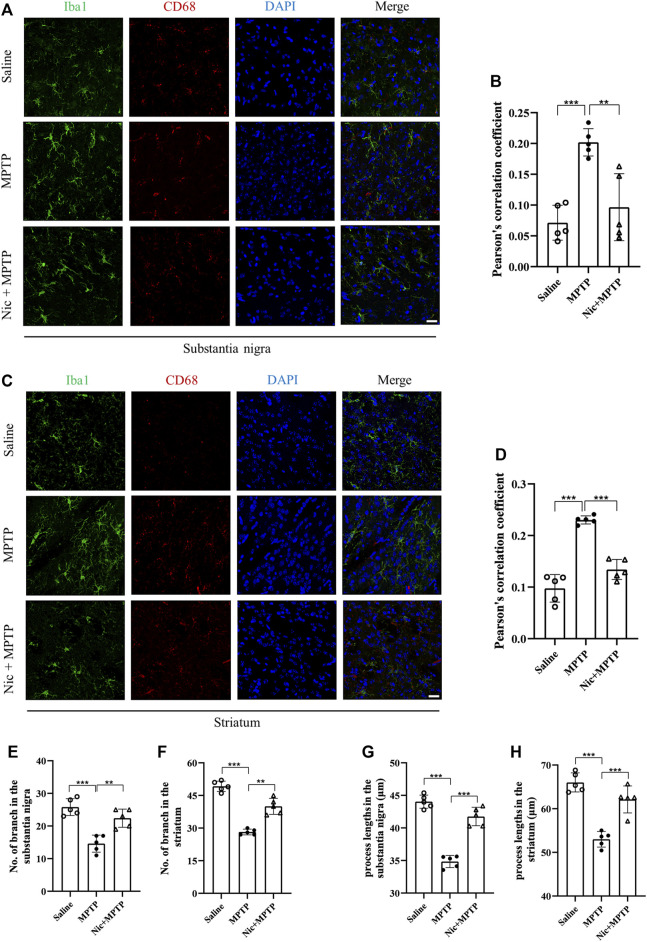
Nicotine inhibits the activation of MPTP-treated mouse substantia nigra and striatal microglia iba1 and the pro-inflammatory factor CD68. **(A)** Immunofluorescence histochemical staining in the substantia nigra. **(B)** Statistical results of colocalization of Iba1 with CD68 in the substantia nigra. **(C)** Immunofluorescence histochemical staining in the striatal regions. **(D)** Statistical results of colocalization of Iba1 with CD68 in the striatal regions. **(E,F)** Statistical results of microglia branching in the substantia nigra and striatal regions. **(G,H)** Statistical results of microglial process lengths in the substantia nigra and striatal regions. Scale bar at 20 μm. Data are expressed as mean ± SEM from five mice in each group, every point on the graph is an average from four fixed fields of view for each mouse. Significant differences were determined by an unpaired Student’s t-test and one-way ANOVA followed by Tukey’s multiple comparison *post hoc* test. *p < 0.05, **p < 0.01, ***p < 0.001.

**FIGURE 5:**
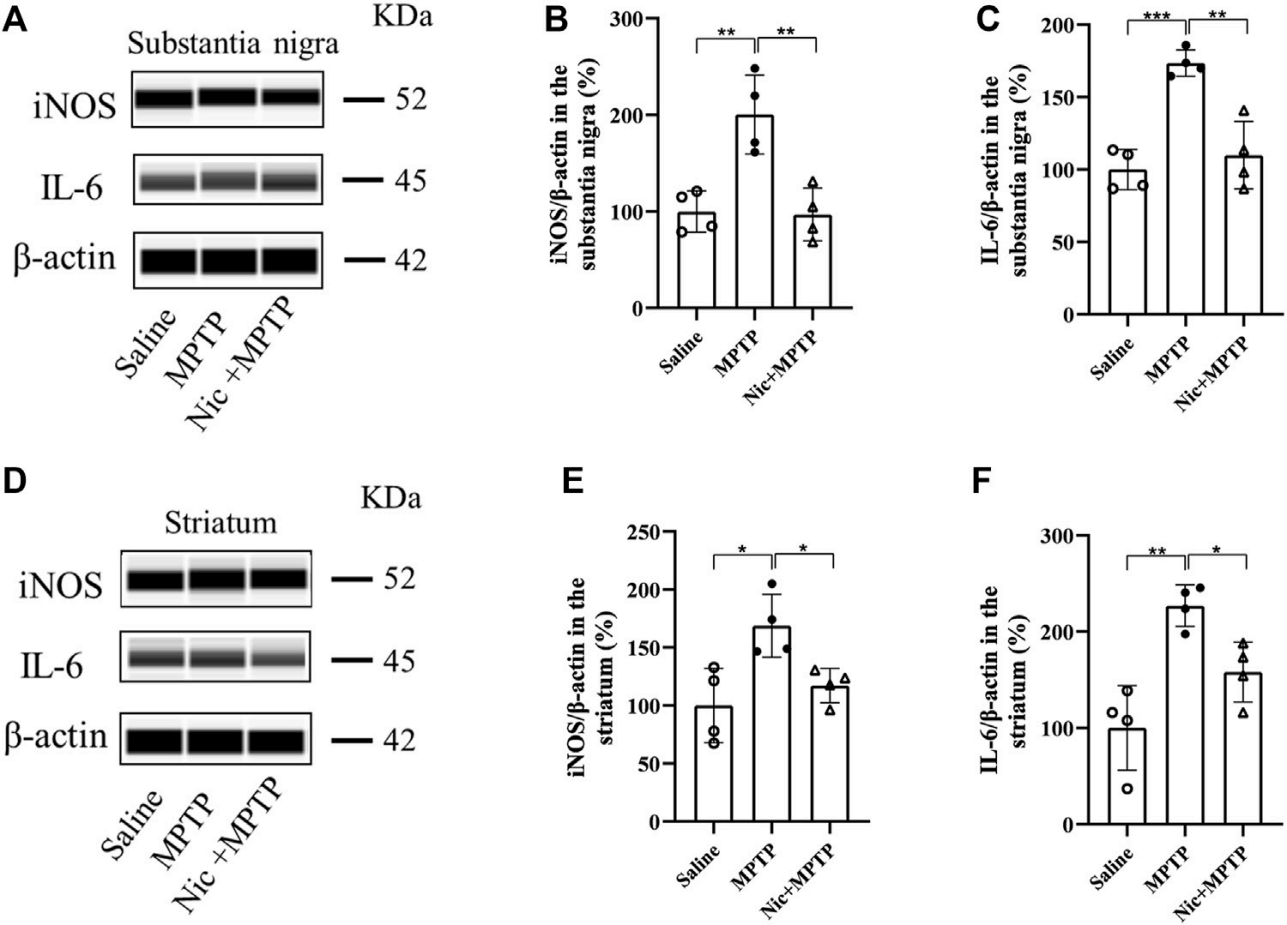
Effect of nicotine on the expression of pro-inflammatory factors iNOS, IL-6 in the substantia nigra and striatum of mptp-treated mice. Representative images and quantification of iNOS, IL-6 immunoblot analysis in substantia nigra **(A)** and striatal tissue **(D)**. **(B,C)** Statistical results of iNOS, IL-6 in the substantia nigra. **(E,F)** Statistical results of iNOS, IL-6 statistics in the striatum. Data are expressed as mean ± SEM from four mice in each group. Significant differences were determined by an unpaired Student’s t-test and one-way ANOVA followed by Tukey’s multiple comparison *post hoc* test. * *p* < 0.05, ** *p* < 0.01, *** *p* < 0.001.

Accumulating evidence reveal that neurodegeneration in PD is usually accompanied by a chronic inflammatory response. To assess the effect of nicotine pretreatment on the inflammatory response in MPTP-induced PD mice models, the expression of microglia-specific protein Iba1, pro-inflammatory factors iNOS and IL-6 were detected by immunofluorescent staining and immunoblot analysis, respectively. We also investigated whether nicotine pretreatment could promote the expression of the M1 microglia by examining the double positive expression of CD68/Iba1 in microglia in different brain regions (Zhou, et al., 2017). Results showed (Figures 4A, C) that the numbers of Iba1-positive microglia and proinflammatory factor CD68 in the substantia nigra and striatum were significantly increased in the MPTP group, with morphologically larger cytosol, clearer contours, more pronounced protrusions and increased number of protrusions. Whereas in the Nic + MPTP group, the Iba1-positive microglia showed ameboid morphology characterized by cell body enlargement, decreased cell branching. Further statistical plots of quantitative analysis of microglial cell morphology data (Figures 4E–H) also showed that the decreased microglial branching and the shortened protrusion in the substantia nigra (*p* < 0.001, *p* < 0.001) and striatum (*p* < 0.001, *p* < 0.001) of the MPTP group, and both of them were significantly increased in the Nic + MPTP group respectively (*p* < 0.01, *p* < 0.01, *p* < 0.001, *p* < 0.001). Colocalization rate of Iba1 and CD68 (Figures 4B, D) was significantly increased in substantia nigra and striatum in MPTP-treated mice (*p* < 0.001, *p* < 0.001), and this was significantly attenuated in the Nic + MPTP group (*p* < 0.01, *p* < 0.001) respectively. These data suggest that nicotine may transform M1 microglial morphology in the substantia nigra and striatum in MPTP-treated mice. To further investigate whether nicotine pretreatment could inhibit the expression of pro-inflammatory factors, we evaluated iNOS and IL-6 expression in the substantia nigra and striatum separately. The results showed (Figures 5A, D) that the expression of iNOS and IL-6 were increased in the substantia nigra and striatum in the MPTP group and both of them were reduced in the Nic + MPTP group, respectively. Further statistical plots (Figures 5B, C, E, F) also showed a significant increase in the expression of iNOS, IL-6 in the substantia nigra (*p* < 0.01, *p* < 0.001) and striatum (*p* < 0.05, *p* < 0.01) in the MPTP group, and Nic treatment could prominently reduce the expression of iNOS, IL-6 (*p* < 0.01, *p* < 0.01, *p* < 0.05, *p* < 0.05). These results indicate that nicotine pretreatment could effectively inhibit the activation of microglia which represents the M1 polarization state and the expression of the pro-inflammatory factor iNOS, IL-6 in the substantia nigra and striatal regions after MPTP stimulation.

### 3.5 Nicotine achieves neuroprotective effects in MPTP induced PD mice by modulating JNK and ERK signaling pathways

**FIGURE 6:**
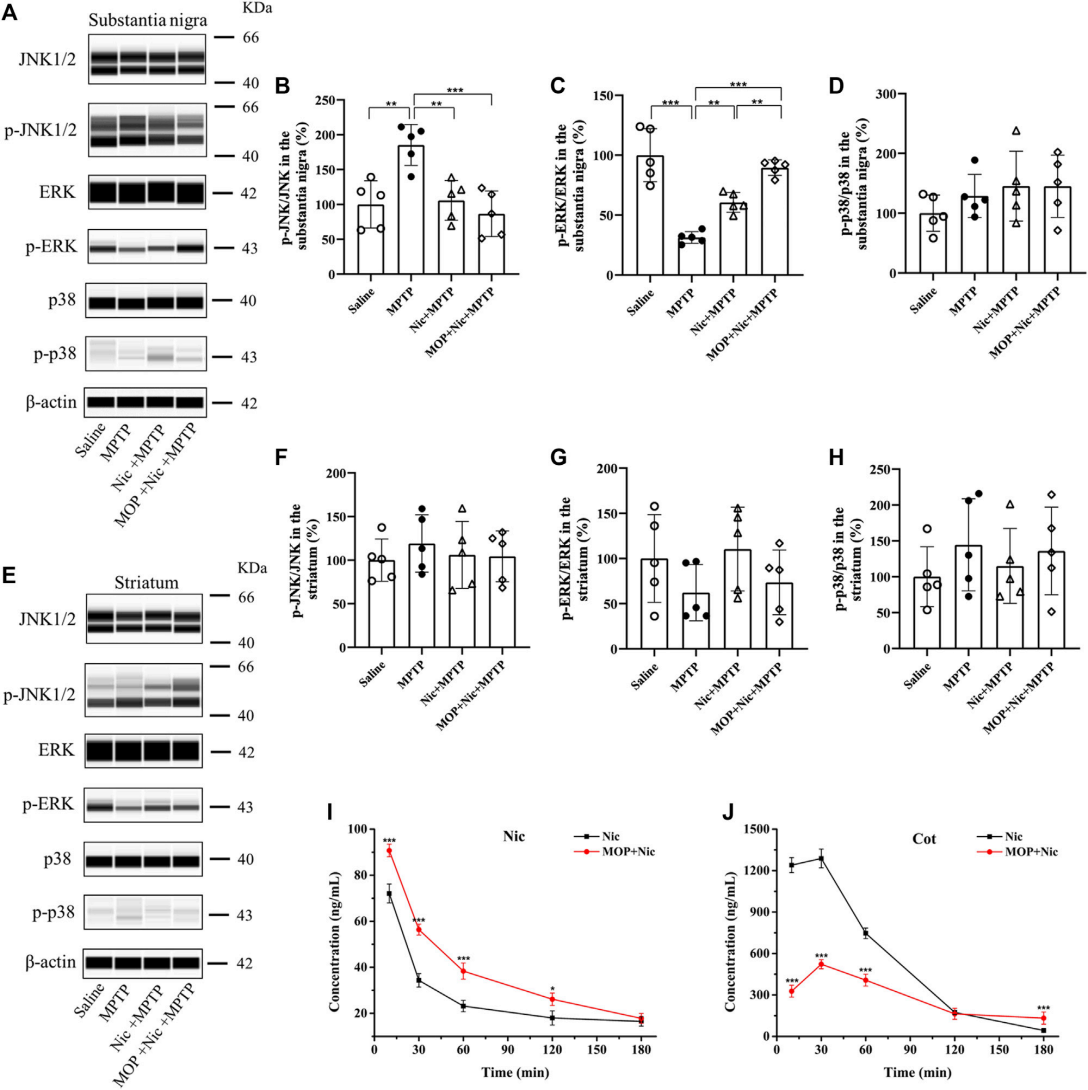
Effects of nicotine on the MPTP-induced expression of MAPK signaling pathways. Representative pictures and quantification for immunoblot analysis of p-JNK/JNK, p-ERK/ERK, p-p38/p38 from the substantia nigra **(A)** and striatal tissues **(E)** are shown. **(B,C,D)** Statistical results of p-JNK/JNK, p-ERK/ERK, p-p38/p38 in the substantia nigra. **(F,G,H)** Statistical results of p-JNK/JNK, p-ERK/ERK, p-p38/p38 statistics in the striatum. **(I,J)** Concentration-time curve of nicotine and cotinine in plasma of saline and MOP-pretreatment mice. Data are expressed as mean ± SEM from four or five mice in each group. Significant differences were determined by an unpaired Student’s t-test and one-way ANOVA followed by Tukey’s multiple comparison *post hoc* test. *p < 0.05, **p < 0.01, ***p < 0.001.

MAPK and related kinase pathways are associated with neurodegenerative diseases including PD, and MAPK signaling pathway plays an important role in cell proliferation apoptosis, differentiation and inflammatory responses, etc. According to previous studies, the mammalian MAPK signaling pathway protein markers (JNK, ERK, p38) were investigated by JESS system to confirm whether Nic achieves neuroprotective effects in MPTP-induced PD mice by modulating the pathway. In addition, as CYP2A5 is an important metabolic enzyme of Nic in mice, we also combinedly introduced the linear furanocoumarin 5-MOP, which is a potent inhibitor of CYP2A5 (Koenigs and Trager, 1998; Lee and Wu, 2005; Damaj, et al., 2007; Alsharari, et al., 2014), to regulate the biodegradation of Nic and further evaluate its effect. The results showed that in the substantia nigra (Figures 6A, B), the MPTP group performed a significant increase in the ratio of p-JNK/JNK (*p* < 0.05), yet Nic pretreatment could efficiently lessen the ratio (*p* < 0.05). MOP-pretreatment inhibited the metabolic process of Nic, which showed that the levels of Nic in the blood of MOP-pretreatment group were higher than that in the saline group within 120 min after administration, and its main metabolite Cot was significantly lower than that in the saline group (Figures 6I, J). Furthermore, in the MOP + Nic + MPTP group the extent of phosphorylation of JNK was even lower compared to the MPTP group (*p* < 0.01) though no significant difference was observed compared with the Nic + MPTP group. Besides, the p-ERK/ERK ratio (Figures 6A, C) was significantly lower (*p* < 0.001) in the MPTP group compared to the saline group in the substantia nigra, nevertheless Nic pretreatment could remarkably increase the p-ERK/ERK ratio (*p* < 0.01), and the modulation of Nic metabolism with MOP could strikingly promote the p-ERK/ERK ratio in comparison with the Nic + MPTP group (*p* < 0.01). However, the p38/MAPK pathway was not efficiently activated in the substantia nigra (Figures 6A, D), as no substantial differences in p-p38/p38 ratio were observed among each group statistically.

Moreover, we also examined the relative mammalian MAPK family proteins in the striatum of mice brains (Figure 6E). Although no significant difference was found in the ratio of p-JNK/JNK, p-ERK/ERK and p-p38/p38 among the groups in the striatum (Figures 6F–H), there is a trend that Nic pretreatment could decline the elevated p-JNK/JNK ratio to control levels, and the inhibition of Nic metabolism was found to further reduce the expression of phosphorylated JNK in MOP + Nic + MPTP group. In brief, these lines of evidence support the idea that Nic achieves neuroprotective effects in MPTP-induced nigrostriatal damage by modulating JNK and ERK signaling pathways.

## 4 Discussion

In patients with PD, neurodegeneration primarily affects dopaminergic neurons in the substantia nigra of the midbrain, leading to progressive loss of nigrostriatal dopaminergic neurons and dysregulation of basal ganglia neuronal circuits (Armstrong and Okun, 2020). This dopaminergic deficit may be the main cause of the locomotor deficit and anxiety-like behavior characteristic of the disease (Zarrindast and Khakpai, 2015). Although repetitive tobacco use could lead to Nic dependence, extensive studies show that Nic exposure appears to confer beneficial effects against PD. Nic is a principal neuroactive ingredient in cigarettes, which can act on the mesolimbic system to produce the reward effect. The neurophysiological and neuropharmacological effects of Nic are mainly due to its high affinity with nAChRs on central neurons and could potently regulate the transmission of various neurochemicals including dopamine. The negative association between Nic intake and PD is mainly attributed to the neuroprotective effect of Nic on nAChRs and dopaminergic neurons damaged by PD (Quik, et al., 2009). Previous studies have shown that Nic reduces nigrostriatal damage and improves motor, cognitive, and memory deficits in PD rodents and monkeys (Parain, et al., 2003; Acharya and Kim, 2021). Also, nicotine may affect anxiety-like behavior and the activation of nAchRs may mediate the anxiolytic-like effects produced by nicotine (Manhaes, et al., 2008; Zarrindast and Khakpai, 2019). And it was reported that specific nAChRs seem to have different roles in the effects of nicotine on anxiety, for example, α7 and α4β2 nAChRs differentially modulate both anxiety and nicotine’s effects on anxiety (Collo, et al., 2018; Oni, et al., 2016; Kutlu, M.G, and Gould, T.J, 2015). In the study, we have found that Nic pretreatment not only could ameliorate MPTP-induced motor deficits and anxiety-like behavior, but also protect against nigrostriatal dopaminergic damage in mice. These results are consistent with the findings of previous research, showing that Nic can effectively ameliorate PD-like symptoms and improve the neuropathological change in the brain of MPTP-induced PD model. However, the detailed cell signaling mechanism of nicotine’s protective effects *in vivo* still calls for elucidation.

Apoptosis is thought to be one of the important mechanisms leading to the death of dopaminergic neurons in the substantia nigra and striatum of PD patients (Schulz, 2006). Therefore, reducing apoptosis and neuronal survival may alleviate the progression of PD. *In vitro* studies have shown that Nic inhibits the mitochondrial apoptotic pathway through the activation of α7-nAChRs, thereby preventing H_2_O_2_-induced apoptosis (Liu, et al., 2015). In the present study, we found that apoptosis was significantly increased in MPTP-induced mice, and Nic pretreatment significantly ameliorated these changes in the substantia nigra and striatal regions. These *in vivo* results suggested that the neuroprotective effects of Nic may be achieved through the regulation of cell apoptosis.

The extensive generation of p-α-syn is an important pathological feature of PD (Fujiwara, et al., 2002; Anderson, et al., 2006; Longhena, et al., 2019). The phosphorylation of α-syn plays an important part in facilitating the formation of the α-syn aggregate species such as oligomers, promoting the Lewy body formation and neurotoxicity (Barrett and Greenamyre, 2015; Cardinale, et al., 2021). In this study, we have found that Nic pretreatment could significantly reduce the elevated p-α-syn in the substantia nigra and striatal dopaminergic neurons of MPTP-treated mice separately. A previous study has shown that Nic can form a transient complex with α-syn, distorting its original structure and altering its aggregation pattern, thereby inhibiting detrimental α-syn oligomer formation (Kardani, et al., 2017). Besides, a recent study was designed to determine whether α7-nAChRs exhibit neuroprotective effects on α-syn pathology and aggregation by promoting α-syn clearance (Zhao, et al., 2021). However, these studies rarely focus on the expression of pathological p-α-syn directly in the nigrostriatal dopaminergic pathway *in vivo*. These findings further support the idea that Nic can reduce the pathological p-α-syn generation in MPTP induced PD model to lessen the dopaminergic neurodegeneration in the substantia nigra and striatum *in vivo*.

Microglia are the largest population of resident immune cells in the brain (Cardinale, et al., 2021), which can be activated after being simulated and play a vital role in inflammation in PD (Liu, et al., 2019). When being activated by the neurotoxin which may damage the CNS, microglia can change their morphology, moving from the “resting” state to the activated state, in with they assume a large cell body and amoeboid shape, with thicker extensions. Moreover, the activated microglia can be mainly divided into M1 phenotype and M2 phenotype, among which, M1 type exhibits amplified levels of pro-inflammatory species, whereas M2 type is anti-inflammatory and expresses anti-inflammation agents (Cardinale, et al., 2021). Therefore, inhibition of abnormal microglia activation is considered as a potential therapeutic strategy for several CNS disorders involving inflammation. Previous studies have shown that Nic exerts anti-inflammatory effects in a variety of cell types and may benefit neurons in various degenerative diseases (Cui and Li, 2010). Studies have reported that CX3CL1, the only member of the CX3C chemokine family, may act as a signal from neurons to glial cells, leading to microglia activation (Lindia, et al., 2005). Recent studies have shown that CX3CL expression is elevated after nicotine exposure, which in turn leads to microglia hyperactivation (Liu, et al., 2020). Activated microglia increase the release of multiple pro-inflammatory cytokines and promote a variety of behaviors (Nakayama, et al., 2019). Our results indicated that Nic pretreatment could effectively inhibit the activation of microglia which represents the M1 polarization state in substantia nigra and striatum after MPTP stimulation. As increasing evidence indicated that drugs targeting pro-inflammatory and pro-killing M1 polarization state could potentially facilitate lessening neuronal damage caused by inflammation (Liu, et al., 2019), Nic may offer a promising alternative therapeutic strategy or participate in the “system protection” in PD. Moreover, further study may focus on whether Nic could induce the phenotype switch from M1 to M2 type and the expression of inflammatory cytokines which may show the therapeutic feasibility for PD.

The activation of nAChR has been reported to trigger the MAPK pathway (Gubbins, et al., 2010; Intskirveli and Metherate, 2012). MAPKs are specific signaling pathways regulating cell differentiation, proliferation, survival, and metabolism. They also play a crucial role in the inflammatory response (Chen, et al., 2016; Odaka, et al., 2016). Nevertheless, whether Nic exerts neuroprotective effects including anti-inflammation *via* MAPK signaling pathway has not been well elucidated *in vivo*. Our present findings seem to be consistent with the previous research, which showed that Nic pretreatment represented neuroprotective anti-apoptotic effects through activation of α7-nAChRs/MAPK/p53 axis *in vitro* (Xu, et al., 2019). Our results showed that in MPTP-treated mice, the expression of p-JNK/JNK in the substantia nigra was significantly increased and the p-ERK/ERK ratio was significantly decreased. It was suggested that Nic could achieve neuroprotective effects in MPTP-induced PD mice by modulating JNK and ERK signaling pathways in the substantia nigra. Even though the striatal regions showed the same trends as those in the substantia nigra, the observed statistical difference in the striatum was not significant. The reason may be because the substantia nigra is more sensitive than the striatal regions, and the striatum is heterogenous (Bjorklund, et al., 2022).

In addition, it has been shown that CYP2A enzymes are responsible for Nic metabolism (Kirby, et al., 2011; Abu-Bakar, et al., 2013; Bagdas, et al., 2014). CYP2A5 is an important enzyme for exogenous compound metabolism in mouse liver, homologous to CYP2A6 in humans, and an important metabolizing enzyme of Nic (Siu, et al., 2006; Raunio, et al., 2008; Zhou, et al., 2010; Li, et al., 2013). The mouse may be a suitable model for studying changes in Nic metabolism and its effects on Nic-dependent mechanisms, including the use of inhibitors to reduce Nic metabolism (Rahnasto, et al., 2005; Damaj, et al., 2007; Visoni, et al., 2008; Alsharari, et al., 2014; Bagdas, et al., 2014). To further observe the neuroprotective effects after modulation of Nic metabolism and disposition in the brain, we further observed the changes of MAPK signaling pathway by using 5-MOP, which is a potent inhibitor of CYP2A5 activity. Our results found that MOP + Nic + MPTP group showed a significant increase in the phosphorylation level of ERK in the substantia nigra region and a further decrease in the phosphorylation level of JNK compared with the Nic + MPTP group. These results indicate that regulation of Nic metabolism may further prolong the neuroprotective effects of Nic. Further research with a greater focus on Nic metabolism with *Cyp2a5*-null mice is suggested. Moreover, the findings indicated that JNK inhibitors may prove effective as a therapeutic medicine for PD and may alleviate the symptoms of PD (Resnick and Fennell, 2004; Kim and Choi, 2010). According to that, Nic may act as a kind of JNK inhibitor for its pharmacological modulation of MPTP induced PD-like symptoms and neuropathological changes in mice.

In summary, Nic pretreatment ameliorates MPTP-induced dyskinesia and anxiety-like behavior in mice with PD. Nic was found to alleviate neuroapoptosis by improving nigrostriatal dopaminergic damage, reducing the accumulation of pathological p-α-syn, and inhibiting microglia activation and pro-inflammatory factor expression in the substantia nigra and striatal regions of mice brain under MPTP stimulation. These neuroprotective effects of Nic may be achieved by modulating the JNK and ERK signaling pathways in the nigrostriatal system, which was further confirmed by the pretreatment of 5-MOP to decline the brain metabolic activity of Nic.

## Data Availability

The original contributions presented in the study are included in the article/supplementary material, further inquiries can be directed to the corresponding authors.
